# NIA Division of Geriatrics and Clinical Gerontology

**DOI:** 10.1093/geroni/igab046.1048

**Published:** 2021-12-17

**Authors:** Evan Hadley

**Affiliations:** National Institute on Aging, Bethesda, Maryland, United States

## Abstract

Dr. Hadley will discuss research priorities for the Division of Geriatrics and Clinical Gerontology. Dr. Hadley will also be available for small group discussion.

